# Supplementary material for: A Synonymous Exonic Splice Silencer Variant in IRF6 as a Novel and Cryptic Cause of Non-Syndromic Cleft Lip and Palate
Source: Genes (Basel). 2020 Aug 7;11(8):903. doi: 10.3390/genes11080903 (PMC7465030; doi:10.3390/genes11080903)
Supplement: Supplementary file 1 [file genes-11-00903-s001.pdf]

Alignment block 1 of 1 in window, 209790628 - 209790652, 25 bps

Links	Species	c.921	
B D	Human	atgaccgctgacctccaggatcagt	
B D	Chimp	atgaccgctgacctccaggatcagt	
B D	Gorilla	atgaccgctgacctccaggatcagt	
B D	Orangutan	atgaccgctgacctccaggatcagt	
B D	Gibbon	atgaccgctgacctccaggatcagt	
B D	Rhesus	gtgaccgctgacctccaggatcagt	
B D	Crab-eating macaque	gtgaccgctgacctccaggatcagt	
B D	Baboon	gtgaccgctgacctccaggatcagt	
B D	Green monkey	atgaccgctgacctccaggatcagt	
B D	Marmoset	gtgaccgctgacctccaggatcagt	
B D	Squirrel monkey	gtgaccgctgacctccaggatcagt	
B D	Bushbaby	gtgaccgctgacctccagaataagt	
	Chinese tree shrew	gtggccgctgacctccaggatcagt	
B D	Squirrel	atgaccgctgacctccaggatcaga	
	Lesser Egyptian jerboa	gtgaccgctgacctccaggatcagt	
	Prairie vole	gtgtccgctgacctccagaatcagt	
B D	Chinese hamster	gtgtccgctgacctccaggatcagt	
	Golden hamster	gtgcccgctgacctccaggatcagt	
B D	Mouse	atgtccgctgacctccaggatcagt	
B D	Rat	atgtccgctgacctccaggatcagt	
B D	Naked mole-rat	atgaccgctgacctccaggatcagt	
B D	Guinea pig	atgaccgctgacctccaggatcagt	
	Chinchilla	atgaccgctgacctccaggatcagt	
	Brush-tailed rat	gtggccgctgacctccaggatcagt	
B D	Rabbit	atgaccgctgacctccaggatcagt	
B D	Pika	atggccgctgacctccaggatcagt	
B D	Pig	gtggccgctgacctccaggatcagt	
B D	Alpaca	atgaccgctgacctccaggatcagt	
	Bactrian camel	atgaccgctgacctccaggatcagt	
B D	Dolphin	atgaccgctgacctccaggatcagt	
	Killer whale	atgaccgctgacctccaggatcagt	
	Tibetan antelope	gtgcccgctgacctccagaatcagt	
B D	Cow	gtgcccgctgacctccaggatcagt	
B D	Sheep	gtgcctgctgacctccagaatcagt	
	Domestic goat	gtgcccgctgacctccagaatcagt	
B D	Horse	gtgaccgctgacctccaggatcagt	
B D	White rhinoceros	gtggccgctgacctccaggatcagt	
B D	Cat	atgaccgctgacctctaggatcagt	
B D	Dog	atgaccgctgacctccaggatcagt	
B D	Ferret	atgaccgctgacctccaggatcagt	
B D	Panda	atgaccgctgacctccaggatcagt	
	Pacific walrus	atgaccgctgacctccaggatcagt	
	Weddell seal	atgaccgctgacctccaggatcagt	
	Black flying-fox	gtgaccgctgacctccaggatcagt	
B D	Megabat	gtgaccgctgacctccaggatcagt	
	Big brown bat	gtgaccgctgacctccaggatcagt	
	David's myotis (bat)	gtgaccgctgacctccaggatcagc	
B D	Microbat	gtgaccgctgacctccaggatcagt	
B D	Hedgehog	gtgcccgctgacctccaggatcagc	

Links	Species	c.921	
B D	Elephant	atgaccgctgacctccaggatcagt	
	Cape elephant shrew	atggccgctgacctccagaatcagt	
B D	Manatee	atgaccgctgacctccaggatcagt	
	Cape golden mole	atgaccgctgacctccaggatcagt	
B D	Tenrec	atgcccgctgacctccaggatcagt	
	Aardvark	atgaccgctgacctcaaggatcagt	
B D	Armadillo	atgaccgctgacctccaggatcagt	
B D	Opossum	atggccactgacttccaagatcagt	*
B D	Tasmanian devil	atggccactgacttccaaaatcagt	*
B D	Wallaby	atggccactgacttccaagatcagt	*
B D	Platypus	atggccgctgacctccaggatcaac	
D	Rock pigeon	atggccactgacctccaggatcagt	
D	Saker falcon	atggccactgacctccaggatcagt	
D	Peregrine falcon	atggccactgacctccaggatcagt	
D	Collared flycatcher	gtggccgctgacctccaggatcagt	
D	White-throated sparrow	gtggccgctgacctccaggatcagt	
B D	Medium ground finch	gtggccgctgacctccaggatcagt	
B D	Zebra finch	gtggccgctgacctccaggatcagt	
	Tibetan ground jay	gtggccgctgacctccaggatcagt	
B D	Budgerigar	atggccactgacctctaggatcagt	
D	Parrot	atggccactgacctccaggatcagt	
D	Scarlet macaw	atggccactgacctccaggatcagt	
D	Mallard duck	atggccactgacctccaggatcagt	
B D	Chicken	atggccactgacctccaggatcagt	
B D	Turkey	atggccactgacctccaggatcagt	
B D	American alligator	atggccactgacctccagaatcagt	
D	Green seaturtle	atggccactgacctccagaatcagt	
D	Painted turtle	atggccactgacctccagaatcagt	
D	Chinese softshell turtle	atggccgctgacctccagaatcagt	
D	Spiny softshell turtle	atggccactgacctccagaatcagt	
B D	Lizard	atggccactgacttctagaatcagc	*
B D	X. tropicalis	atgcccgctgacctctagtatcagg	
B D	Coelacanth	atggccattgacttccagaataagt	*
B D	Tetraodon	atggccgctgacctccaggatcaga	
B D	Fugu	gtggccactgacctccaggatcaaa	
	Yellowbelly pufferfish	gtggccactgacctccaggatcaaa	
B D	Nile tilapia	gtggccgctgacctctaggatcaga	
	Princess of Burundi	gtggccgctgacctccaggatcaga	
	Burton's mouthbreeder	gtggccgctgacctccaggatcaga	
	Zebra mbuna	gtggccgctgacctccaggatcaga	
	Pundamilia nyererei	gtggccgctgacctccaggatcaga	
B D	Medaka	atgtccgctgacctcaagaatgagg	
	Southern platyfish	gtggccgctgatctccaggatcaga	*
B D	Stickleback	gtgaccgctgacctccaggatgagg	
B D	Atlantic cod	gtggccgctgacctccaggatcagc	
B D	Zebrafish	gtgcccgctgacctccaggatgagc	
	Mexican tetra (cavefish)	ctgccctcggatctccaggatcagc	*
	Spotted gar	gtgtccgctgacctccaggatgagg	
